# Monitoring prolactin in patients taking antipsychotics

**DOI:** 10.3389/fpsyt.2024.1333280

**Published:** 2024-01-30

**Authors:** Aria Ghahramani, Alfredo Bellon

**Affiliations:** ^1^Department of Psychiatry and Behavioral Health, Penn State Hershey Medical Center, Hershey, PA, United States; ^2^Department of Pharmacology, Penn State Hershey Medical Center, Hershey, PA, United States

**Keywords:** antipsychotics, prolactin, schizophrenia, bipolar disorder, depression, osteopoenia/osteoporosis, sexual dysfuntion, endocrinology

Despite the benefits of antipsychotic medications, there are known and severe side effects. The cardiometabolic side effects have been well-studied, and there are practice guidelines directing their management (1). In contrast, while most antipsychotics are known to increase prolactin levels, routine monitoring of serum prolactin has not yet been adopted as standard practice. The Clinical Guidelines of The Endocrine Society recommend monitoring of prolactin in symptomatic patients, and in certain pathologic states, however, they do not comment on how to proceed when patients are prescribed medications that are known to increase prolactin levels (2). Given the negative consequences of hyperprolactinemia (3), and the possibility for reasonable modifications to antipsychotic regimens (4, 5), we suggest routine monitoring of prolactin in patients who are prescribed antipsychotics.

The current standard in psychiatric practice is not to measure serum prolactin unless the patient becomes symptomatic, but hyperprolactinemia may be asymptomatic in the short term, and in the long term, there is potential for serious sequelae. In men and women, undetected hyperprolactinemia over time may cause decreased energy, reduced muscle mass, infertility, osteopenia, and bone fractures (6, 7).

Differences between prolactin effects among antipsychotics have been well-studied, with Amisulpride and Risperidone causing the greatest increase in serum prolactin, and Aripiprazole decreasing prolactin levels. The adverse effects are generally dose-dependent, and differential effects are thought to be mostly due to varying degrees of dopamine-2-receptor antagonism (8).

Given the serious side-effects of hyperprolactinemia and the possibilities for changing management by lowering dosage or switching to a different antipsychotic, we propose routinely monitoring prolactin levels in patients who are prescribed antipsychotics. A baseline prolactin level should be drawn prior to initiation of antipsychotics, and a follow-up level after titration is completed. While this may add to the cost of healthcare (9), we believe that the benefits to patients outweighs the financial burden. Just as we monitor cardiac and metabolic effects of antipsychotics, prolactin monitoring can help us provide more thoughtful and patient-centered treatment.

## Author contributions

AG: Investigation, Writing – original draft. AB: Conceptualization, Supervision, Writing – review & editing.
